# First insights into the shortfin mako shark (*Isurus oxyrinchus*) fine-scale swimming behaviour

**DOI:** 10.1098/rsos.230012

**Published:** 2023-05-03

**Authors:** Bruno M. Saraiva, Bruno C. L. Macena, Silvio Solleliet-Ferreira, Pedro Afonso, Jorge Fontes

**Affiliations:** ^1^ Ocean Sciences Institute2014;OKEANOS, University of the Azores, Rua Professor Doutor Frederico Machado 4, 9901-862 Horta, Portugal; ^2^ Institue of Marine Research, University of the Azores, Rua Professor Doutor Frederico Machado 4, 9901-862 Horta, Portugal

**Keywords:** biologging, buoyancy, energetics, burst swimming, gliding

## Abstract

As regional endotherms, lamnid sharks can sustain high cruising speeds and perform frequent speed bursts. However, since endothermy comes with high energetic costs, lamnids may adopt different swimming strategies to manage their energy budget. Understanding such strategies is essential to provide behavioural and physiological context to their broader movement ecology. The endangered shortfin mako (*Isurus oxyrinchus*) possibly has the highest energy requirements among lamnids, but our understanding of its swimming behaviour is still limited. We equipped three shortfin mako sharks with high-resolution multi-sensor tags to measure their swimming kinematics in the wild. While swimming horizontally, individuals favoured tail-beat frequencies around 0.6 Hz at speeds comparable to those of ectothermic sharks (*ca* 0.5 m s^−1^). All individuals displayed yo-yo-like diving patterns where, for a given tail-beat frequency, speeds were higher during descents, as expected for a negatively buoyant fish. Contrary to what was expected, gliding was almost absent (less than 1.31%). Speed bursts reaching up to 3.6 m s^−1^ were observed during the day but ceased shortly after dusk, implying a diel change in swimming behaviour. As large-scale research efforts are hindered by this species' increasing rarity, opportunistic high-resolution datasets, like the present, are fundamental to improve our understanding of shortfin mako's behaviour and ecology.

## 1. Introduction

Like tunas and billfishes, lamnid sharks (or mackerel sharks) possess remarkable morphological and physiological adaptations enhancing their swimming performance. Such adaptations include the centralization of the slow-twitch, oxidative myotomal muscle (i.e. red muscle, RM) and a specialized counter-current heat exchange system (rete mirabile) to and from the RM, brain, eyes and viscera, allowing them to conserve metabolic heat and maintain these body regions above ambient temperatures, a capacity commonly known as regional endothermy [1,2].

Regional endothermy is frequently coupled with a higher aerobic swimming metabolism, which, together with the ability to maintain elevated internal temperatures, might enable lamnid sharks to have an increased potential for burst swimming and sustain higher cruising speeds that, in turn, may increase prey encounter rates and enhance their ability to identify and capture prey [3–5]. However, an elevated aerobic swimming metabolism increases energy expenditure. Thus, these animals are likely to adopt appropriate swimming strategies to manage their energy surplus over time [6,7].

Understanding such strategies is crucial to provide behavioural and physiological context to these sharks' large-scale movements. Nevertheless, this knowledge is still limited due to the many obstacles involved in studying large, highly mobile animals in the wild. Due to recent advances in multi-sensor biologging technologies (accelerometers, magnetometers, gyroscopes, speed sensors, etc.), we can now start to comprehend fundamental aspects of animal behaviour through quantitative measurements of their body kinematics [6,8–10]. Recent multi-sensor biologging studies, on endothermic and ectothermic sharks, have shown that sharks' swimming patterns are probably connected to behaviours that minimize energy expenditure while maximizing foraging opportunities [7,10,11].

Within the Lamnidae family, the shortfin mako shark (*Isurus oxyrinchus*, hereafter referred to as the mako shark) stands out as one of the fastest marine fishes [12]. However, the mako shark's exceptional swimming capacity results in extreme oxygen consumption rates [13] and particularly high energetic requirements [14]. This species is distributed across tropical and temperate oceans, from approximately 60° N to 50° S, showing a preference for higher latitudes [15–17]. In the North Atlantic, the mako shark is known to aggregate within the highest fishing risk zone, with an estimated space use overlap of 62% with longline fisheries [17]. As a result of excessive catch rates [18] and highly k-selected life-history traits (i.e. slow growth rate, late sexual maturity, low fecundity) [16,19], the mako shark is currently recognized as globally endangered by the International Union for the Conservation of Nature's Red List [20]. Therefore, improving our understanding of the mako shark's swimming behaviour is of utmost importance but, to our knowledge, there are still no *in situ* studies concerning this subject.

Here, we describe the first high-resolution dataset obtained from multi-sensor biologging tags of mako sharks tracked in the Azores, mid-North Atlantic. Our study provides direct measurements of mako shark's swimming speeds and tail-beat frequencies, along with the first insights into this species' fine-scale swimming behaviour.

## 2. Material and methods

### 2.1. Shark tagging

Tag deployments were conducted in July 2020 near Faial Island (38.60° N, 28.50° W), in the Azores archipelago, Portugal. Three mako sharks were tagged with G-Pilot tags [21] using a non-invasive harness-like towing system [22]. Different deployment methods were used for each shark: shark #01, a 143 cm female, was captured using longlines (3 h of soaking time); shark #02, a 155 cm male, was caught with a fishing rod after being attracted with chum (*ca* 15 min of handling); shark #03, a 160 cm female, was attracted with chum and tagged while freely swimming following [22]. All sharks were tagged in the water, with tagging procedures ranging from a few seconds, for shark #03, to 7 min, for sharks #01 and #02. The sharks' total lengths were measured to the nearest centimetre or visually estimated in relation to the boat, for captured and free-swimming sharks, respectively (table 1).

**Table 1.** Summary of tagged shortfin mako sharks (*Isurus oxyrinchus*). TL, total length; F, female; M, male.

shark ID	date	latitude (°)	longitude (°)	duration	TL (cm)	sex	tagging
#01	14 July 2020	38.53927	–28.96008	14 h 41 min	143	F	longline
#02	20 July 2020	38.52697	−29.01528	14 h 5 min	155	M	fishing rod
#03	22 July 2020	38.62308	−28.54305	34 min	160	F	free-diving

G-Pilot multi-sensor packages were equipped with a tri-axial accelerometer, a tri-axial gyroscope, depth and temperature sensors measuring at 20 Hz, and a tri-axial magnetometer programmed to record at 100 Hz in order to allow speed estimations (see Swimming speed section below for details). In addition, each package also included a SPLASH10-F tag (ARGOS-link with FastLockGPS, Wildlife Computers, Inc. WA, USA) and a VHF radio transmitter (F1835B, Advanced Telemetry Systems, Inc. MN, USA). After release from the sharks, the tags were recovered using a combination of ARGOS locations and radio telemetry [21], and their data were offloaded for analysis. No tagging apparel was left on the animals.

### 2.2. Swimming metrics

Tri-axial sensor data were initially visualized using the Igor Pro software v. 6.22 (Wavemetrics, Inc. LO, USA) with the Ethographer add-in [23] to extract basic swimming metrics. Subsequent analyses were conducted in R v. 4.1.3 [24].

Using the surging acceleration for sharks #02 and #03 and the sway acceleration for shark #01, time-series of dominant tail-beat frequencies (TBF) and amplitudes at 1 Hz resolution were extracted from spectrum analysis based on a continuous wavelet transformation (CWT). Although it has been shown that surging acceleration provides a clearer tail-beat signal for the G-Pilot towed tag system [21], we used the swaying acceleration for shark #01 due to a failure of the accelerometer recording longitudinal acceleration. Visual comparisons of surge and sway accelerations from sharks #02 and #03 showed similar patterns (electronic supplementary material, figure S1 and figure S2). Furthermore, employing the same method described above, TBF and amplitudes were derived using surge angular velocity data (longitudinal axis) measured by the tag's gyroscope and compared with those estimated from acceleration.

Since angular velocity produced a clearer tail-beat signal, it was used to detect gliding events. We employed the k-mean algorithm to cluster the behavioural spectra according to differences in frequency. A spectrum with no peaks was considered to represent gliding events [25]. The fit of the resulting cluster was then visually inspected against the surging angular velocity signal (i.e. creating a mask corresponding to the gliding cluster overlaid to the surging angular velocity plot) and manually corrected (i.e. comparing the mask with the angular velocity signal and removing the parts that did not correspond to gliding behaviour). As cluster analysis from shark #02 was not successful, possibly due to the extremely low incidence of gliding behaviour, glides for this individual were manually identified.

### 2.3. Swimming speed

The swimming speeds of sharks #01 and #02 were estimated from the magnetic paddle wheel rotation frequency recorded by the tags' magnetometer. The spinning frequencies of the eight magnets (paddles) for each 1 s interval were extracted from spectrum analysis and then converted to the animals' swimming speeds using a predetermined correlation between rotation frequency (in Hz) and drop speed (in m s^−1^) for each G-Pilot package. Hence, matching the final resolution of estimated tail-beat frequencies (1 Hz).

In short, to calibrate the rotation frequency, the tags were vertically dropped multiple times with varying weights. The vertical velocities from each drop were then linearly regressed against the respective average paddle wheel's rotation frequency [21]. The resulting regression models explained more than 99% of the vertical velocity's variance (electronic supplementary material, figure S3). A sampling rate of 100 Hz was chosen to allow the magnetometer sensor to read the paddle wheel magnets passing at high speed [21].

### 2.4. Recovery period

We used the tail-beat cycle (i.e. the inverse of tail-beat frequency) to estimate the recovery period after tagging, employing an adaptation of the methods described in [26]. In summary, the tail-beat cycle was averaged in 15 min intervals and plotted against post-release time, revealing a significant asymptotic relation (electronic supplementary material, figure S4). The time elapsed until the average tail-beat cycle reached 80% of its right horizontal asymptote was considered the recovery period. Asymptotic models were fit with the R package drc v. 3.0-1 [27] using a self-started three-parameter mean function. Given the short track (34 min) of shark #03, the recovery period was not determined for this individual.

### 2.5. Diving behaviour

To classify phases within dives, we initially smoothed the depth trace using a 10 s running mean. Vertical velocities (VV) were then calculated employing the central difference of the smoothed depth over 1 s windows. Ascents and descents were defined as intervals with a VV lower than −0.05 m s^−1^ and higher than 0.05 m s^−1^, respectively, for over 1 min. Swimming was considered horizontal when the absolute value of VV was below 0.05 m s^−1^ for at least 1 min. Periods in which the dive phases were not stable (lasting less than 1 min) were classified as transient phases. Mean dive angles of the two sharks (#01 and #02) with speed measurements were estimated from track sections with small fluctuations in VV. The distance travelled during the selected dives was first determined using each dive's average swimming speed. Dive angles were then obtained by applying the arcsine function to the dive's vertical displacement divided by the actual distance travelled by the shark. Ascent and descent angles were compared using a non-parametric Wilcoxon rank-sum test. Since sharks might present different diving patterns that maximize their energy surplus according to the circumstances, the longest dives performed by sharks #01 and #02 (vertical extent greater than 80 m; electronic supplementary material, figure S5) were used to visually identify optimum swimming strategies based on [28] analytically generated models. Additionally, to detect burst swimming events, we used the double of the modal swimming speed observed for each shark as a threshold, separately calculated for each recovery phase.

## 3. Results

### 3.1. Tagging performance and recovery period

**Figure 1.**
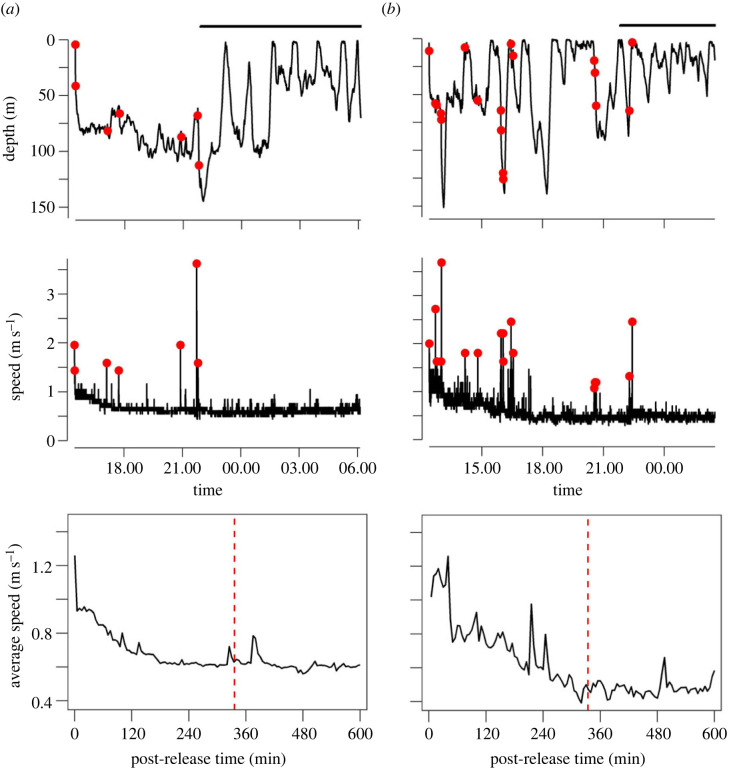
Full time-series of the depth profile (top), speed estimates (middle), and respective average speeds (5 min fixed windows) during the first 600 min (10 h) after release (bottom) from shortfin mako sharks (*Isurus oxyrinchus*) #01 (*a*) and #02 (*b*). Red circles indicate burst swimming events. Top horizontal black lines denote the local night-time period. Vertical dashed red lines indicate the recovery time estimated from the tail-beat cycle.

Three mako sharks were tagged in this study (table 1): sharks #01 and #02 were tracked for over 14 h each, whereas shark #03 tag was prematurely released after 34 min. Sharks #01 and #02 displayed higher tail-beat activity immediately after tagging, gradually decreasing throughout the recovery period. Based on the mean tail-beat cycle, we estimated a recovery period of 336 min for shark #01 and 334 min for shark #02 (electronic supplementary material, figure S4). An analogous trend was apparent in the swimming speeds of both individuals, with initial swimming speeds *ca* two to four times higher than post-recovery cruising speeds (figure 1).

### 3.2. Swimming performance

**Figure 2.**
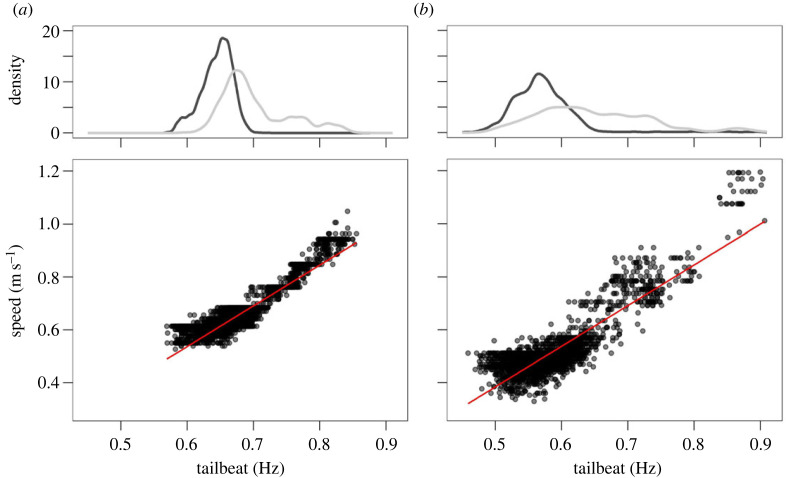
Tail-beat frequencies from shortfin mako sharks (*Isurus oxyrinchus*) #01 (*a*) and #02 (*b*) during horizontal swimming. Density distributions (top) of experienced tail-beat frequencies colour-coded by recovery period (pre-recovery, light grey; post-recovery, dark grey). Linear relationship (bottom) between average tail-beat frequencies and average swimming speeds (red lines) determined for each 5 s fixed window after removing observations with spectrum entropies above 90% (shark #01 *R*^2^ ± 0.83; shark #02 *R*^2^ ± 0.76).

During periods of horizontal swimming, the average swimming speeds of sharks #01 and #02 showed a strong linear relationship with average TBF (figure 2). All sharks tracked in this study displayed right-tailed distributions of TBF, with the most used TBF *ca* 0.60 Hz corresponding to a speed of *ca* 0.53 m s^−1^ while horizontally swimming. Modal TBF during horizontal swimming observed for shark #01 was 0.67 Hz post-release and 0.66 Hz after recovery. Shark #02 displayed smaller modal TBF both before and after its recovery, 0.58 and 0.56 Hz, respectively. The modal TBF exhibited by shark #03 while horizontally swimming was 0.61 Hz (table 2).

**Table 2.** Summary statistics calculated for the entire dataset and for each shortfin mako shark (*Isurus oxyrinchus*). TBF, tail-beat frequency; VV, vertical velocity.

	overall	Shark #01			Shark #02			Shark #03
		pre-recovery	post-recovery	overall	pre-recovery	post-recovery	overall	overall
mean depth (m)	52.14 ± 36.25	83.99 ± 12.35	56.55 ± 36.54	68.14 ± 31.71	49.72 ± 32.92	27.89 ± 29.35	38.37 ± 32.41	54.92 ± 47.77
maximum depth (m)	151.36	109.00	144.65	144.65	151.36	139.08	151.36	134.01
mean temperature (°C)	24.11 ± 2.27	21.60 ± 0.53	23.47 ± 1.78	22.76 ± 1.70	24.65 ± 1.80	26.05 ± 1.77	25.49 ± 1.90	24.73 ± 2.44
temperature range (°C)	20.80–28.57	20.99–27.71	20.88–26.77	20.88–27.71	21.26–28.57	21.10–27.82	21.10–28.57	20.80–27.78
mean TBF (Hz)	0.65 ± 0.09	0.71 ± 0.07	0.64 ± 0.05	0.67 ± 0.07	0.69 ± 0.12	0.58 ± 0.06	0.62 ± 0.10	0.67 ± 0.11
TBF range (Hz)	0.25–2.00	0.57–2.00	0.25–2.00	0.25–2.00	0.30–1.08	0.35–0.90	0.30–1.08	0.31–1.05
mean ascent TBF (Hz)	0.70 ± 0.07	0.70 ± 0.02	0.69 ± 0.03	0.69 ± 0.03	0.75 ± 0.08	0.66 ± 0.05	0.69 ± 0.08	0.73 ± 0.09
mean descent TBF (Hz)	0.57 ± 0.11	0.74 ± 0.17	0.57 ± 0.06	0.59 ± 0.10	0.60 ± 0.14	0.50 ± 0.04	0.54 ± 0.11	0.68 ± 0.17
mean horizontal TBF (Hz)	0.64 ± 0.07	0.70 ± 0.05	0.64 ± 0.02	0.67 ± 0.05	0.65 ± 0.09	0.57 ± 0.03	0.58 ± 0.06	0.61 ± 0.04
mean ascent VV (m s^−1^)	0.12 ± 0.06	0.07 ± 0.01	0.09 ± 0.03	0.09 ± 0.03	0.14 ± 0.06	0.12 ± 0.05	0.12 ± 0.53	0.26 ± 0.12
mean descent VV (m s^−1^)	0.13 ± 0.19	0.18 ± 0.18	0.11 ± 0.06	0.12 ± 0.09	0.14 ± 0.16	0.9 ± 0.05	0. ± 0.3	0.63 ± 0.83
mean speed (m s^−1^)	0.63 ± 0.16	0.71 ± 0.14	0.62 ± 0.08	0.66 ± 0.11	0.73 ± 0.24	0.50 ± 0.07	0.59 ± 0.20	—
speed range (m s^−1^)	0.29–3.69	0.49–1.96	0.44–3.62	0.44–3.62	0.33–3.69	0.29–2.45	0.29–3.69	—
mean ascent speed (m s^−1^)	0.60 ± 0.10	0.66 ± 0.05	0.63 ± 0.04	0.63 ± 0.04	0.66 ± 012	0.52 ± 0.07	0.58 ± 0.12	—
mean descent speed (m s^−1^)	0.66 ± 0.25	0.94 ± 0.33	0.64 ± 0.10	0.68 ± 0.18	0.79 ± 0.37	0.52 ± 0.11	0.64 ± 0.29	—
mean horizontal speed (m s^−1^)	0.60 ± 0.12	0.69 ± 0.10	0.61 ± 0.03	0.65 ± 0.08	0.63 ± 0.118	0.48 ± 0.05	0.51 ± 0.11	—
no. burst swimming events	25	5	2	7	13	5	18	—
mean ascent angle (°)	10.99 ± 4.07	—	—	7.38 ± 2.55	—	—	13.09 ± 3.30	—
mean descent angle (°)	8.68 ± 2.64	—	—	9.09 ± 2.77	—	—	8.37 ± 2.62	—
gliding %	0.23	0.06	0.30	0.35	0.06	0.01	0.07	1.31
maximum glide time (s)	49	15	49	49	10	4	10	13

**Figure 3.**
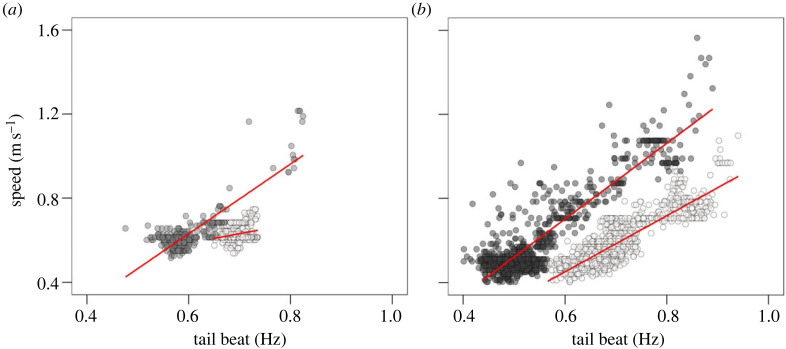
Tail-beat frequencies during vertical movements from shortfin mako sharks (*Isurus oxyrinchus*) #01 (*a*) and #02 (*b*) colour-coded by dive phase (ascents, white circles; descents, dark grey circles). Linear relationship between average tail-beat frequencies and average swimming speeds (red lines) calculated for each 5 s fixed window after removing observations with spectrum entropies above 90% was separately determined for each dive phase (shark #01: ascent *R*^2^ ± 0.07, descent *R*^2^ ± 0.70; shark #02: ascent *R*^2^ ± 0.77, descent *R*^2^ ± 0.80).

A significant linear relationship between average swimming speeds and average TBF was found for sharks #01 and #02, both during ascents and descents (figure 3). However, the shark #01 ascent model exhibited a low percentage of explained variance (*R*^2^ ± 0.07), as the observed TBF was centred around 0.7 Hz with little variability across the TBF range observed for the species. The slope of the shark #02 descent model was considerably steeper than the one of its ascent model. Moreover, for a given TBF, both sharks #01 and #02 displayed higher swimming speeds during descents than ascents. Similarly, shark #03 average TBF was higher during ascents than descents (table 2).

Shark #01 exhibited the highest TBF recorded throughout the study, occasionally reaching 2 Hz both before and post-recovery, while sharks #02 and #03 swam at a maximum of 1.08 Hz and 1.05 Hz TBF, respectively (table 2). After the recovery period, sharks #01 and #02 only exceeded 0.80 Hz for brief periods. Shark #03 exhibited an identical behaviour only 6 min post-tagging, suggesting a considerably faster recovery period compared with the two captured sharks.

**Figure 4.**
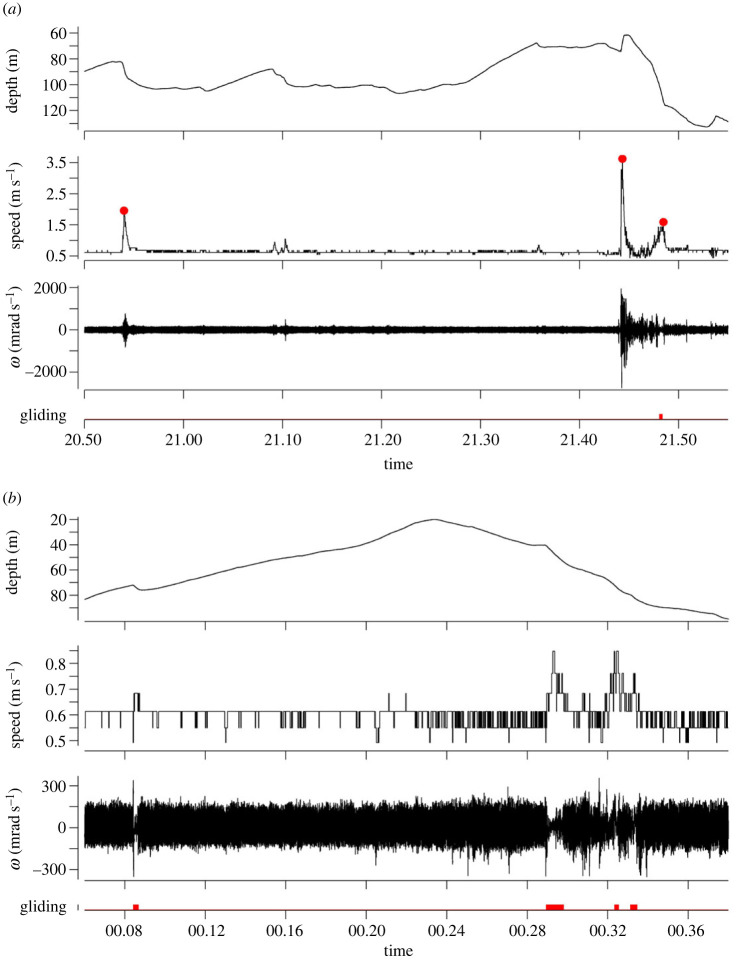
Two subsections of shortfin mako shark (*Isurus oxyrinchus*) #01 (*a,b*) time-series displaying depth, speed, surge angular velocity (***ω***) and identified gliding events (red mask). Red circles mark speed bursts.

We detected a total of 25 burst swimming events associated with peaks in TBF, with shark #02 showing the largest number of swimming bursts, including the maximum speed recorded in this study (3.69 m s^−1^) (table 2). Burst swimming events were detected for the two individuals before and after recovery through all dive phases (i.e. ascent, descent and transient phases) except horizontal swimming, usually directed downwards (93%—positive VV) but two upward-directed movements (6%—negative VV) of which the steepest was performed by shark #01 (figures 1 and 4*a*). Only 4% of the burst events occurred during ascent phases with the remaining 96% equally distributed during descent and transient phases. Burst events were not observed after nightfall (figure 1).

### 3.3. Swimming strategies

All tagged mako sharks exhibited a yo-yo-like diving behaviour favouring deeper waters during the day (figure 1). Although present, gliding was an infrequent behaviour recorded sparsely and typically as brief events, usually following a peak in surging angular velocity (figure 4*a,b*) and associated with positive vertical velocities (i.e. descents). This relatively low occurrence of gliding behaviour resulted in a very small percentage of time spent gliding (0.07 to 1.31%). The longest glide recorded during this study was performed by shark #01 (49 s) (table 2). Furthermore, asymmetrical diving was observed throughout the shark #02 track, with ascents being marginally steeper than descents (Wilcoxon rank-sum test *p*-value = 0.0023). Additionally, 81.82% of shark #02 descents were flatter than the corresponding ascents. By contrast, ascent and descent angles identified for shark #01 were not significatively different (Wilcoxon rank-sum test *p*-value = 0.2991), with 57.14% of descents being steeper than subsequent ascents (table 2 for diving angle statistics).

Moreover, six out of the seven selected dives from sharks #01 and #02 agreed with analytically generated optimal swimming strategies explored in [28]. Most of these dives (five) were consistent with an ‘intentional' diving strategy led by VV, where swimming speed increases with absolute VV, referred to by the authors as ‘type 2' (electronic supplementary material, figure S6). Still, one dive from shark #02 was identified as ‘type 1’ (electronic supplementary material, figure S7), where propulsive power alternates between low and high (i.e. slow descents and fast ascents). This behaviour is possibly connected to a ‘resting' strategy.

## 4. Discussion

This study provides a small yet unique high-resolution dataset on the diving kinematics and swimming speeds of three free-swimming mako sharks. Our results open a new window into the swimming behaviour of this iconic, endangered, apex predator of the open ocean while presenting the research potential of minimally invasive tagging tools as a future standardized protocol that can be widely used in this type of study.

We estimate a recovery period of roughly 5.5 h for sharks #01 and #02 after their release, during which a gradual decline in swimming speeds was observed. Previous studies using capture and release methods with other obligate ram ventilators have described identical recovery patterns [6,29]. Sharks #01 and #02 may have shifted from aerobic to anaerobic metabolism during the strenuous process of fishing, restraining and tagging, thus consuming their glycogen stocks and accumulating lactate [30,31]. One way to reduce lactate accumulation is via oxidation, which may be achieved faster by increasing the active metabolic rate and oxygen uptake (e.g. by elevating swimming speeds immediately after release) [29]. Although shark #03 track was quite short, its low post-tagging sustained swimming activity (lower TBF) suggests that the non-invasive tagging [22] had minimal impacts on the animal's behaviour and energetics, presumably because no additional lactate was produced during tagging and handling procedures.

It has been shown that regional endotherms cruise roughly 1.6 times faster than their ectothermic counterparts [3], with the mako shark being considered the fastest of all sharks [12]. Earlier indirect estimates using short-term acoustic tracking (2 to 45 h) revealed average cruising speeds ranging from 0.70 to 1.86 m s^−1^ with maximum speeds reaching 9.10 m s^−1^ [32,33]. In our study, mako sharks typically swam at substantially slower speeds (0.50 to 0.62 m s^−1^ [0.32 to 0.43 TL s^−1^]), identical to those of ectothermic sharks [4,34]. Likewise, recorded burst speeds were comparable to those described for ectothermic oceanic whitetip (*Carcharhinus longimanus*) and grey reef (*Carcharhinus amblyrhynchos*) sharks [10,35]. Moreover, previous TBF records based on visual observations were considerably higher (1.00 to 1.10 Hz) than ours (overall 0.58 to 0.71 Hz; post-recovery average less than 0.64 Hz). Yet, the sharks in that study were much smaller than our free-swimming sharks (95 to 105 cm versus 143 to 160 cm) [13]. Therefore, discrepancies in TBF and swimming speeds might be associated with methodological, environmental, or size-related differences (mechanic or behavioural) [36,37].

All individuals tagged in our study displayed a yo-yo-like swimming strategy. This behaviour was present both before and after the recovery period of sharks #01 and #02, suggesting that it was not caused by capturing or handling stress. Previous studies have described identical yo-yo swimming patterns in large pelagic fishes [6,7,25], yet the motivations behind such behaviour are not fully understood. It has been proposed that yo-yo diving may be related to behavioural thermoregulation, navigation, foraging and energetically efficient swimming [8,25,38,39].

Weihs [38] theorized that negatively buoyant fish could attain over 50% energy savings by combining powerless gliding during gravity-assisted descents with active swimming ascents. Our results confirm that mako sharks are negatively buoyant since swimming speeds were faster during descents than ascents using the same TBF. Likewise, TBF had a stronger positive effect on swimming speeds during descents. However, contrary to expectations, tagged individuals rarely performed gliding behaviour, with glides accounting only for 0.07 to 1.31% of the whole tracks. Infrequent gliding was previously described for other species like the tiger shark (*Galeocerdo cuvier*) (less than 18%) [25], the blue shark (10 to 20%) [8], and the Greenland sleeper shark (*Somniosus microcephalus*) (0.2 to 12%) [40]; however, compared with the mako sharks in our study these species glide consistently.

Weihs' model [38] also predicts shallower dive angles during unpowered descents than during powered ascents. Shark #02 diving patterns were consistent with this assumption, but ascent and descent angles from shark #01 were not significatively different, with most descents being indeed steeper than corresponding ascents. The same pattern observed for shark #01 was described for seven mako sharks tracked throughout the Southern California Bight [33], suggesting that the asymmetrical diving strategy is not often used by this species.

One explanation for the paucity of gliding behaviour could be that the cessation of propulsive strokes would result in reduced metabolic activity, which could lead to a decrease in the mako shark's internal temperature during descents, with possible detrimental effects on swimming performance. On the other hand, while some pelagic sharks, such as blue and oceanic whitetip sharks, have long and wide pectoral fins designed to promote lift, the bulky torpedo-like body and short fins of the mako shark are tailored for speed but not ideal for gliding [1,10,41]. Hence, the rarity of gliding behaviour may simply be a consequence of the mako shark's morphology. Nonetheless, larger sharks have bigger livers (i.e. greater lipid storages), which may provide closer to neutral buoyancies, probably influencing the animals' swimming performance [41,42]. Future studies should target larger animals to determine the effect of body size on swimming strategies before more robust generalizations can be made.

Pelagic predators are expected to select swimming strategies that maximize their energy surplus while searching for prey. Our mako sharks' diving patterns were consistent with this assumption, with most dives selected to identify optimum swimming strategies supporting an ‘intentional' diving strategy, probably associated with prey-searching behaviour [28]. Burst swimming events, which possibly represent predation attempts or successes [8,9], were identified for sharks #01 and #02 almost exclusively during the daytime, when the sharks were yo-yo swimming within a wider portion of the water column, suggesting that this strategy may maximize prey encounter rates. Although most of the observed speed bursts were directed downwards, a sudden steep high-speed upward-directed movement (3.62 m s^−1^) was recorded for shark #01. Such a pattern is probably associated with an ambush hunting strategy, as mako sharks might take advantage of their countershading and burst swimming capabilities to ambush their prey [43,44].

We cannot exclude the possibility that these high-energy events represent an escape response to larger predators or even a reaction to the towed tags but, given that speed bursts stopped shortly after dusk, the last hypothesis seems unlikely. The absence of speed bursts at night suggests a change in foraging strategy or a decrease in prey availability. For instance, if mako sharks partially rely on upward ambushing, then it is likely that this strategy is exclusive to daytime, at least in the absence of strong moonlighting. A previous study using stomach temperatures to identify feeding events in mako sharks has described the same pattern, where five of the six feeding events occurred during daytime [33].

Even if based on a limited dataset, given the paucity of data on mako sharks' swimming speeds and diving kinematics, the present findings have significant implications for our understanding of this key species’ behaviour and energetics and should be accounted for in future research and conservation efforts. Notwithstanding, our results must be considered cautiously. Sharks might display distinct levels of behavioural plasticity varying across environmental landscapes, ontogenetically and individually [41,45,46]. Therefore, additional high-resolution datasets are needed before more robust generalizations can be made. Future large-scale research efforts will be hampered by the increasing scarcity of this endangered oceanic predator. New opportunistic small datasets, such as the one presented here, will be essential to improve our understanding of mako sharks' behaviour and ecology in order to support urgent management and population recovery plans.

## Data Availability

The datasets supporting this article have been uploaded as part of the electronic supplementary material [47].
